# Socio-demographic factors associated with COVID-19 vaccine uptake and refusal among Ugandan women

**DOI:** 10.1186/s12992-023-00968-z

**Published:** 2023-09-06

**Authors:** Andreas Backhaus

**Affiliations:** Federal Institute for Population Research (BiB), Wiesbaden, Germany

**Keywords:** COVID-19, Vaccine acceptance, Vaccine hesitancy, Gender

## Abstract

**Background:**

This paper analyzes associations of socio-demographic factors with the uptake of COVID-19 vaccines, the refusal to get vaccinated against COVID-19, and various reasons stated for refusing vaccination against COVID-19 among a representative sample of Ugandan women.

**Methods:**

This paper utilizes a representative cross-sectional survey collected among women aged 15-49 years in Uganda between September and November 2021. Regression analyses are used to study the associations of a broad range of socio-demographic characteristics with COVID-19 vaccine uptake, refusal of vaccination, and reasons for refusal among the respondents.

**Results:**

4211 women were included in the analysis. 11% of them were vaccinated against COVID-19, 76% were willing to get vaccinated, 13% were unwilling to get vaccinated. Fear of side effects was the most commonly stated reason for refusing vaccination (69%). Factors significantly and positively associated with being vaccinated against COVID-19 were age, higher education, urban residency, having savings, partial instead of complete income loss during the pandemic, and usage of modern contraceptives. Factors significantly and positively associated with refusing vaccination against COVID-19 were urban residency and current pregnancy, while age, having savings, and using modern contraceptives were factors associated with a lower likelihood of refusing vaccination, albeit with varying statistical significance. Few factors were strongly related to the stated reasons for refusing the vaccines; the fear of side effects significantly increased with age, while having received negative information on the vaccines was significantly less common among women with higher education.

**Conclusions:**

This study documents a low COVID-19 vaccination rate and a high willingness to get vaccinated among Ugandan women. Positive age and education gradients in vaccine uptake point to inequity in access to vaccination, potentially resulting from prioritizations of groups at particularly high risk. Refusal to be vaccinated was relatively low and systematic factors behind vaccine refusal were hardly to be found, even less so for particular reasons given for refusal.

## Introduction

Global inequity in vaccination against COVID-19 has been high, resulting in substantial health and economic costs for low-vaccinated countries, with the latter remaining disproportionately located in sub-Saharan Africa [1, 2]. Furthermore, vaccine hesitancy and refusal in sub-Saharan Africa have been brought into focus as obstacles to achieving wide-spread immunization against COVID-19 [3, 4]. However, the evidence is still scarce regarding actual vaccine uptake and refusal among different socio-demographic strata of African societies.

This study contributes to the understanding of COVID-19 vaccine uptake and refusal in sub-Saharan Africa by analyzing socio-demographic factors associated with the COVID-19 vaccination decisions of women in Uganda. The survey underlying this study features several favorable characteristics: First, it asked whether the surveyed Ugandan women had been vaccinated against COVID-19. Second, it asked those women who had not been vaccinated yet about their intentions regarding vaccination against COVID-19 in the future. Third, it asked those women who responded they would be unwilling to be vaccinated against COVID-19 in the future to state their reason(s) for refusing vaccination. This information is complemented by socio-demographic characteristics of the survey population.

**Fig. 1:**
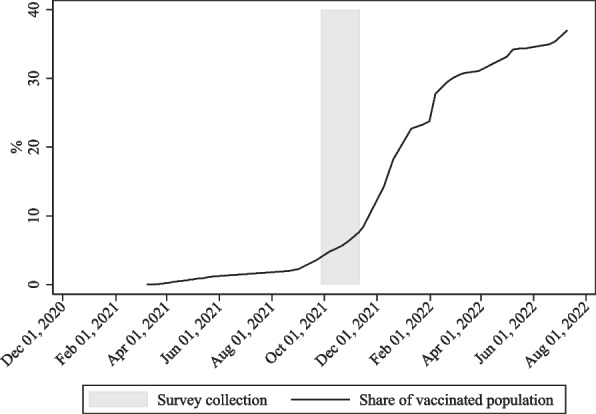
Progress of vaccination against COVID-19 in Uganda. *Note:* The figure displays the vaccination rate against COVID-19 of the Ugandan population over time. The shaded area indicates the period of the survey collection. *Source:* Author’s own depiction based on data provided by Our World In Data and PMA [6, 7]

**Fig. 2:**
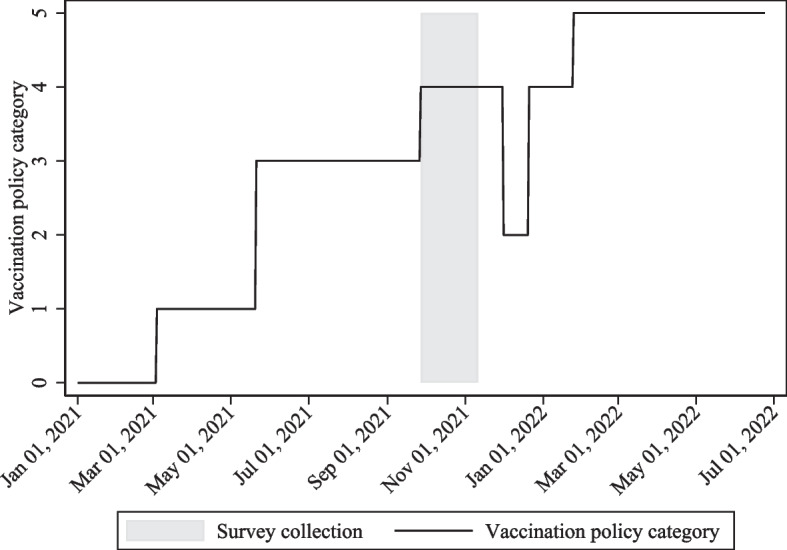
COVID-19 vaccination policies in Uganda. *Note:* The figure displays the COVID-19 vaccination policy in Uganda over time. Category 0 indicates that no vaccines are available. Category 1 indicates that vaccines are available for ONE of the following: key workers/ clinically vulnerable groups / elderly groups. Category 2 indicates that vaccines are available for TWO of the following: key workers/ clinically vulnerable groups / elderly groups. Category 3 indicates that vaccines are available for ALL the following: key workers/ clinically vulnerable groups / elderly groups. Category 4 indicates that vaccines are available for all three, plus partial additional availability (selected broad groups/ages). Category 5 indicates universal availability of vaccines. The shaded area indicates the period of the survey collection. *Source:* Author’s own depiction based on data provided by Our World In Data, OxCGRT and PMA [6–8]

The context of vaccination against COVID-19 in Uganda, which is classified as a low-income country by the World Bank [5], has been similar to the experience of other sub-Saharan African countries to the effect that Uganda’s vaccination campaign has been hampered by the lack of vaccine doses. Figure 1 displays the progress of vaccination against COVID-19 in Uganda, with the shaded area indicating the period in 2021 when the survey underlying this study was collected. During the survey collection, Uganda’s vaccination campaign accelerated, with the share of the Ugandan population that had received at least one dose of any COVID-19 vaccine rising from below 5% in late September 2021 to 10% by mid-November 2021. The survey collection further closely coincided with an expanded availability of the COVID-19 vaccines in Uganda, as displayed in Fig. 2: While the vaccines had previously only been available to key workers, clinically vulnerable groups and elderly groups, as indicated by category 3 of the vaccination policy indicator, it further became available to broader age groups in late September 2021.

Similar to the state of knowledge on other sub-Saharan African countries, previous studies on COVID-19 vaccination in Uganda have been focused on the *hypothetical* uptake of COVID-19 vaccines once the latter become available: When surveying the willingness to participate in COVID-19 vaccine trials, 70.2% of 657 Ugandan healthcare workers were willing to participate in such trials in 2020 [9]. Surveys further reported a stated acceptance rate of the COVID-19 vaccines of 70.1% among 317 Ugandan individuals at increased risk for severe COVID-19 disease [10], of 53.6% among 1067 predominantly male respondents in Western Uganda [11], of only 37.3% among 600 Ugandan medical students [12], of more than 75% in two subnational surveys targeting women in 13 Ugandan districts and residents of the capital Kampala, respectively [13], and of 57.8% among 1053 Ugandan adults [14]. A multi-country survey of 15 African countries found a comparatively high stated acceptance rate of 87% for the COVID-19 vaccines among 1008 Ugandan respondents [15]. This study therefore complements and expands the existing evidence by analyzing representative data on actual uptake, refusal and reasons for refusal.

## Methods

### Survey design

The data for this study was collected and published by the Performance Monitoring for Action (PMA) project [7]. Primarily, the PMA Uganda Phase 2 Household and Female survey collected data on knowledge, practice, and coverage of family planning services among women aged 15-49 in 141 enumeration areas (EAs) selected using a multi-stage stratified cluster design with urban-rural strata. The survey is representative at the national level of Uganda and within urban/rural strata. The data was collected between September and November 2021. Further details of the survey design and sampling procedure are described in the accompanying user notes [16].

Importantly, this particular phase of the PMA survey also asked whether women had been vaccinated against COVID-19 and, if they had not been vaccinated yet, whether they would be willing to be vaccinated against COVID-19 in the future if vaccines became available to them. Women who stated that they were not willing to be vaccinated against COVID-19 were further asked to state their reason(s) for their refusal of the vaccination.

### Survey variables

The survey design allows differentiating the responding women into three groups: Those that have been vaccinated against COVID-19, those that have not been vaccinated yet but were willing to be vaccinated, and those who were unwilling to be vaccinated. The purpose of the following analysis was threefold: First, it was to understand which socio-demographic factors were associated with having been vaccinated. Second, it was to understand which socio-demographic factors were associated with being unwilling to be vaccinated. Third, it was to analyze the socio-demographic factors associated with particular reasons stated for vaccine refusal. Consequently, the survey responses referring to the COVID-19 vaccination status and inclination were coded into 1) a binary indicator equal to unity if the responding woman has been vaccinated against COVID-19, and zero otherwise, and 2) a binary indicator equal to unity if the responding woman was unwilling to be vaccinated against COVID-19 in the future, and zero otherwise. Nine distinct reasons for refusal of vaccination were pre-specified and recorded by the survey, with an additional category collecting other, rare mentions. A binary indicator was coded for each of the nine reasons; each indicator was set to unity if a woman who was unwilling to be vaccinated selected this particular reason, and set to zero otherwise. Respondents could select multiple reasons for vaccine refusal.

The socio-demographic factors that were included as explanatory variables in the regression analysis were: age grouped into four categories (15-19, 20-29, 30-39, 40-49 years), highest level of schooling attended and grouped into three categories (primary or less, any secondary, tertiary or higher), relative household wealth grouped into three tertiles (lowest, middle, highest), residential status (rural or urban), whether the respondent had recently worked, whether the respondent had savings, the respondent’s household’s income loss during the pandemic (partial or complete), whether the respondent had given birth in 2020/21 or was currently pregnant, and whether the respondent was using modern or traditional methods of contraception. Relative household wealth was based not on income but on a household wealth score computed by combining information on a household’s assets, livestock owned, floor, roof, and wall construction materials, water sources, and toilet facilities, following the methodology of the wealth index used for the Demographic and Health Surveys (DHS) [17].

### Statistical analysis

All statistical analyses of the data were performed in Stata Version 16.0. Inverse probability weights were applied in the computation of all descriptive statistics and regression results reported in the following. The associations of the socio-demographic factors with the COVID-19 vaccination outcomes were estimated as linear probability models by regressing each of the two binary vaccination outcomes on the socio-demographic factors. The associations of the socio-demographic factors with the stated reasons for vaccine refusal were estimated by regressing the binary indicator for each stated reason on the socio-demographic factors. Standard errors were robust to heteroskedasticity, while tables report *p*-values in parentheses underneath each coefficient estimate. Estimated coefficients were considered to be statistically significant only if $P \le 0.05$.

## Results

### Descriptive statistics on the respondents

**Fig. 3:**
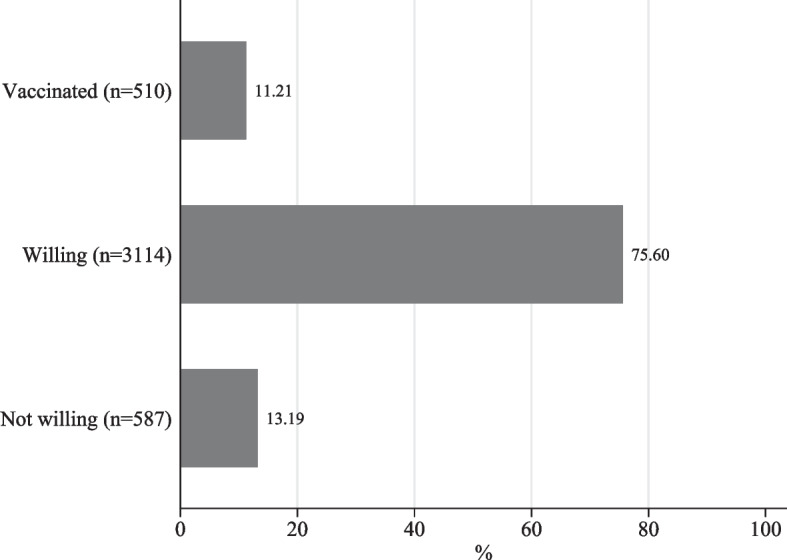
COVID-19 vaccine uptake and vaccination intentions among the surveyed women in Uganda. *Note:* The figure displays the number of surveyed Ugandan women who are vaccinated against COVID-19 (top bar), who are unvaccinated but willing to get vaccinated against COVID-19 (middle bar) and who are not willing to get vaccinated against COVID-19 (bottom bar). The corresponding percentages are displayed at the end of each bar. *Source:* Author’s own depiction. Sampling weights are applied

Table 1 presents summary statistics on the sample of Ugandan women that were included in the analysis. 60% of the female respondents were younger than 30 years and 63% had only attended primary schooling or no schooling at all. 22% lived in urban areas, 50% had worked recently, and 41% had savings. 35% of them reported that their households had experienced a complete instead of only a partial income loss since the beginning of the pandemic. 27% had given birth in 2020 or 2021, while 9% were pregnant by the time of the survey collection. 35% were users of modern contraceptives, while only 6% used traditional ones. Figure 3 displays the totals and shares of unvaccinated and vaccinated sample respondents, respectively. At the time of the survey collection, only 11% of the surveyed Ugandan women were already vaccinated against COVID-19, while a corresponding share of 89% was not vaccinated. Figure 3 further shows that a large majority of 76% among the surveyed women stated that they were willing to get vaccinated once a vaccine would be available to them. 13% of the surveyed women expressed that they would not get vaccinated even once vaccination was available.

**Table 1:** Descriptive statistics of the sample of surveyed women in Uganda

Variable	Freq.	Mean
Age groups:		
Age 15-19	1007/4211	0.24
Age 20-29	1530/4211	0.36
Age 30-39	1055/4211	0.26
Age 40-49	619/4211	0.15
Highest level of schooling attended:		
Primary education or less	2607/4211	0.63
Any secondary education	1273/4211	0.30
Tertiary or higher education	331/4211	0.07
Relative household wealth:		
Lowest wealth tertile	1298/4211	0.31
Middle wealth tertile	1337/4211	0.33
Highest wealth tertile	1576/4211	0.36
Rural or urban residence:		
Urban	1489/4211	0.22
Economic characteristics:		
Has worked recently	2112/4211	0.50
Any savings	1750/4211	0.41
Complete instead partial income loss since pandemic	1399/4211	0.35
Fertility:		
Given birth in 2020/21	1071/4211	0.27
Currently pregnant	367/4211	0.09
Contraceptive usage:		
Using modern contraceptives	1447/4211	0.35
Using traditional contraceptives	255/4211	0.06
Vaccination outcomes:		
Vaccinated against COVID-19	510/4211	0.11
Willing to be vaccinated	3114/4211	0.76
Unwilling to be vaccinated	587/4211	0.13

Note: The middle column displays frequencies of each variable, while the righthand column displays weighted sample means.

Source: Author’s own computation

### Factors associated with COVID-19 vaccine uptake

Table 2 presents the results of regression analyses investigating socio-demographic factors associated with COVID-19 vaccine uptake among the female Ugandan survey population. Using age group 15-19 as the baseline, the coefficients reported in column 1 indicate a significantly higher probability of being vaccinated for all three older age groups, with the magnitudes slightly increasing from 6.1 percentage points (pp) for age group 20-29 to 7.7 pp for age group 40-49. Column 1 further reports results regarding the association between education and COVID-19 vaccine uptake: Relative to the baseline of women with primary or less than primary education, women with any secondary education were 2.4 pp more likely to be vaccinated; however, the coefficient was only marginally statistically significant. Both magnitude and significance of the association increased when considering women with tertiary or other higher education, as the latter were significantly more likely to be vaccinated, with the magnitude of the coefficient (16.1 pp) being sizable relative to the overall vaccination rate. Household wealth, in turn, was not significantly associated with the probability of being vaccinated, as women whose households belonged to the middle or the highest wealth tertile were not more likely to be vaccinated than women whose household belonged to the lowest wealth tertile. Women living in urban areas of Uganda were 3.3 pp more likely to be vaccinated than women living in rural areas, with the difference being statistically significant. All of these associations were robust regarding their magnitude and significance across the four different specifications reported in this table.

**Table 2:** A multivariate linear regression showing factors associated with having been vaccinated against COVID-19 among women in Uganda

	(1)	(2)	(3)	(4)
**Age**				
Age 15-19	omitted	omitted	omitted	omitted
	(.)	(.)	(.)	(.)
Age 20-29	0.061	0.054	0.068	0.053
	(0.000)	(0.000)	(0.000)	(0.000)
Age 30-39	0.066	0.056	0.070	0.057
	(0.000)	(0.000)	(0.000)	(0.000)
Age 40-49	0.077	0.066	0.074	0.070
	(0.000)	(0.000)	(0.000)	(0.000)
Primary education or less	omitted	omitted	omitted	omitted
	(.)	(.)	(.)	(.)
Any secondary education	0.024	0.022	0.023	0.024
	(0.068)	(0.090)	(0.078)	(0.070)
Tertiary or higher education	0.161	0.149	0.159	0.164
	(0.000)	(0.000)	(0.000)	(0.000)
Lowest wealth tertile	omitted	omitted	omitted	omitted
	(.)	(.)	(.)	(.)
Middle wealth tertile	0.005	0.008	0.004	0.004
	(0.690)	(0.543)	(0.731)	(0.742)
Highest wealth tertile	0.021	0.022	0.020	0.020
	(0.152)	(0.132)	(0.175)	(0.172)
Rural	omitted	omitted	omitted	omitted
	(.)	(.)	(.)	(.)
Urban	0.033	0.034	0.032	0.032
	(0.042)	(0.034)	(0.053)	(0.045)
Has not worked recently		omitted		
		(.)		
Has worked recently		0.022		
		(0.058)		
No savings		omitted		
		(.)		
Has savings		0.043		
		(0.001)		
Partial income loss		omitted		
		(.)		
Complete income loss		-0.025		
		(0.037)		
No birth in 2020/21			omitted	
			(.)	
Birth in 2020/21			-0.020	
			(0.156)	
Not pregnant			omitted	
			(.)	
Pregnant			-0.019	
			(0.340)	
Not using modern contraceptives				omitted
				(.)
Using modern contraceptives				0.032
				(0.016)
Not using traditional contraceptives				omitted
				(.)
Using traditional contraceptives				-0.010
				(0.666)
N	4,211	4,211	4,211	4,211

Note: *P*-values reported in parentheses.

Source: Author’s own computation

Column 2 reports results from further adding indicators of economic activity to the regression. Women who had supplied labor besides domestic work in the past 7 days were 2.2 pp more likely to be vaccinated than women who had not. Further, women who had savings were on average 4.3 pp more likely to be vaccinated than women who did not have savings. In turn, women whose household had experienced a complete loss of income due to the pandemic were 2.5 pp less likely to be vaccinated than women whose household had only experience a partial loss of income. Each of the three coefficients was at least weakly statistically significant.

In turn, two fertility-related variables were not associated with the vaccine uptake, as reported in column 3: Neither recent births in 2020 or 2021 nor a current pregnancy were significantly related to the probability of being vaccinated. Interestingly, as reported in column 4, women who used modern contraceptive were 3.2 pp more likely to be vaccinated than women who did not use modern contraceptives. This significant association did not translate to women who were users of traditional contraceptives.

### Factors associated with COVID-19 vaccine refusal

Table 3 reports the results of regression analyses investigating socio-demographic factors associated with COVID-19 vaccine refusal among the surveyed Ugandan women. Relative to the age group 15-19, women in higher age groups were less likely to refuse vaccination against COVID-19; however, the difference was statistically significant across specifications only for women aged 20-29, who were between 3.7 and 5.4 pp less likely to refuse vaccination, while it was small and insignificant for women aged 30-39 in every regression. Women in age group 40-49 were between 2.7 and 3.6 pp less likely to refuse vaccination, but the difference was weakly significant in one specification only. Notably, the probability of refusing vaccination was not significantly different among women with secondary or tertiary and higher education than among women with primary education or less. Women whose households belonged to the middle wealth tertile were nearly 3 pp less likely to refuse vaccination than women whose households belonged to the lowest wealth tertile, with the difference being at least weakly significant in all specifications, while it was insignificant for women whose households belonged to the highest wealth tertile. Women living in urban areas of Uganda were significantly more likely to refuse vaccination, with the magnitude of the association varying between 3.4 and 3.9 pp across specifications.

**Table 3:** A multivariate linear regression showing factors associated with refusing COVID-19 vaccination among women in Uganda

	(1)	(2)	(3)	(4)
**Age**				
Age 15-19	omitted	omitted	omitted	omitted
	(.)	(.)	(.)	(.)
Age 20-29	-0.047	-0.042	-0.054	-0.037
	(0.004)	(0.010)	(0.002)	(0.030)
Age 30-39	-0.020	-0.014	-0.023	-0.008
	(0.277)	(0.453)	(0.222)	(0.672)
Age 40-49	-0.036	-0.029	-0.032	-0.027
	(0.082)	(0.172)	(0.115)	(0.201)
Primary education or less	omitted	omitted	omitted	omitted
	(.)	(.)	(.)	(.)
Any secondary education	-0.009	-0.006	-0.008	-0.008
	(0.523)	(0.656)	(0.593)	(0.569)
Tertiary or higher education	0.021	0.025	0.022	0.020
	(0.452)	(0.365)	(0.423)	(0.479)
Lowest wealth tertile	omitted	omitted	omitted	omitted
	(.)	(.)	(.)	(.)
Middle wealth tertile	-0.029	-0.025	-0.028	-0.028
	(0.049)	(0.088)	(0.051)	(0.058)
Highest wealth tertile	-0.022	-0.019	-0.021	-0.020
	(0.211)	(0.276)	(0.226)	(0.236)
Rural	omitted	omitted	omitted	omitted
	(.)	(.)	(.)	(.)
Urban	0.037	0.034	0.039	0.039
	(0.018)	(0.028)	(0.014)	(0.013)
Has not worked recently		omitted		
		(.)		
Has worked recently		0.017		
		(0.185)		
No savings		omitted		
		(.)		
Has savings		-0.029		
		(0.025)		
Partial income loss		omitted		
		(.)		
Complete income loss		-0.014		
		(0.296)		
No birth in 2020/21			omitted	
			(.)	
Birth in 2020/21			0.009	
			(0.532)	
Not pregnant			omitted	
			(.)	
Pregnant			0.056	
			(0.020)	
Not using modern contraceptives				omitted
				(.)
Using modern contraceptives				-0.031
				(0.024)
Not using traditional contraceptives				omitted
				(.)
Using traditional contraceptives				-0.039
				(0.114)
N	4,211	4,211	4,211	4,211

Note: *P*-values reported in parentheses.

Source: Author’s own computation

As reported in column 2, recent labor supply by the surveyed women was not associated with their probability of refusing vaccination, while having savings was significantly associated with a 2.9 pp lower probability of refusing vaccination. Women whose households had experienced a complete income loss did not differ significantly in their likelihood of refusing vaccination from women whose households had experienced only a partial income loss during the pandemic.

While a recent birth event was not associated with vaccine refusal, a current pregnancy was significantly associated with a 5.6 pp higher probability of refusing vaccination among Ugandan women (column 3).

As shown in column 4, Ugandan women who were using modern contraceptives were on average 3.1 pp less likely to refuse vaccination, which was a statistically significant difference to women who were not using modern contraceptives. Women who were using traditional methods of contraception were also less likely to refuse vaccination, while the difference of 3.9 pp to women who were not using traditional methods was not significant.

### Factors associated with reasons for refusing COVID-19 vaccination

**Fig. 4:**
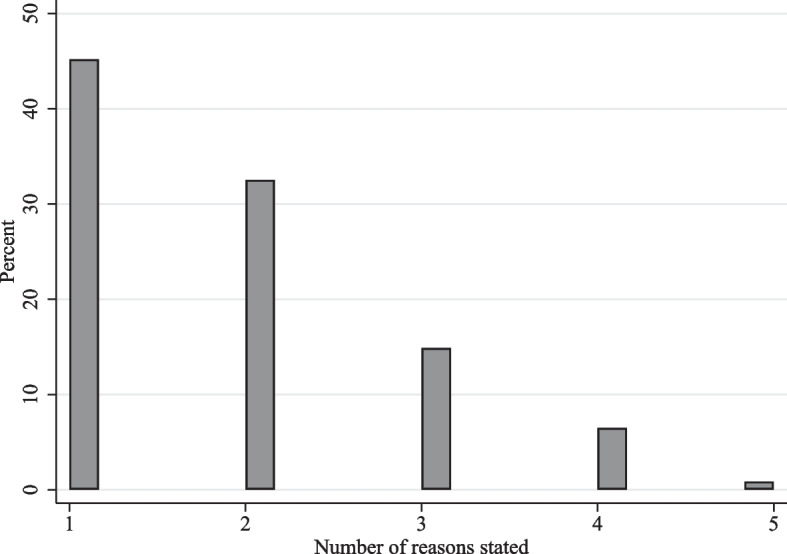
Multiplicity of reasons for refusing vaccination against COVID-19 among the surveyed women in Uganda. *Note:* The figure displays a histogram of the number of reasons selected by surveyed Ugandan women in order to explain their refusal of vaccination against COVID-19. *Source:* Author’s own depiction

Recall that respondents who refused vaccination against COVID-19 could select from nine pre-specified reasons for their refusal, with the possibility of selecting multiple reasons and with reasons outside the pre-specified ones being collected in a tenth response item. Among the 587 vaccine-refusing Ugandan women in the sample, only 3 did not state any reason for refusal; these observations were omitted from the analyses reported in the following. Among the remaining 584 vaccine-refusing women, 264 or about 45% selected a single reason for their refusal, while a majority of 320 or about 55% selected multiple reasons. Complementarily, Fig. 4 displays the relative multiplicity of reasons stated for refusing vaccination against COVID-19. While about one-third of the refusing respondents selected two reasons, three or more reasons were selected considerably less frequently, suggesting that respondents who refused vaccination were able to pin down specific reasons for their refusal.

Table 4 further informs on the relative importance of the various pre-specified reasons for vaccine refusal. 69% of the women who refused vaccination against COVID-19 agreed that they did so due to their fear of side effects from the vaccines. This was by far the most frequently selected reason for vaccine refusal. 32% of the refusing respondents further selected that they questioned the effectiveness of the vaccines. 26% of the refusing respondents agreed that they had received negative information about the vaccines via the media, while 16% agreed that they lacked comprehensive information about the vaccines. Fear of pain was selected by 10% as a reason for refusal, while 9% selected a lack of belief in vaccines. 6% agreed that they were unwilling to get vaccinated because they did not consider themselves to be at risk of COVID-19. Only 2% selected religious opposition to vaccines as a reason for vaccine refusal, while only 1% agreed that their vaccine of choice was not available in Uganda. 12% of the refusing respondents stated other reasons.

**Table 4:** Descriptive statistics of reasons for vaccine hesitancy among surveyed women in Uganda

Reason for refusal	Freq.	Share
Fear of side effects/ health related concerns	411/584	0.69
Doubt over vaccine effectiveness	194/584	0.32
Lack of comprehensive information about the vaccines	93/584	0.16
Negative information in the media	143/584	0.26
Not at risk of COVID-19	40/584	0.06
Doesn’t believe in vaccines	54/584	0.09
No vaccine of choice in the country	7/584	0.01
Fear of pain	68/584	0.10
Religious opposition	14/584	0.02
Other reason	58/584	0.12

Note: The middle column displays frequencies of each variable, while the righthand column displays weighted sample means.

Source: Author’s own computation

Table 5 reports regression results on associations between socio-demographic characteristics of Ugandan women who refused vaccination against COVID-19 and the particular reasons stated for the refusal. Overall, only few variables correlated significantly and systematically with one or several reasons for refusing vaccination. The fear of side effects from vaccination was significantly and substantially elevated among women in the age groups 20-29 and 40-49 compared to women in the age group 15-19, while women whose households belonged to the middle wealth tertile exhibited a weakly significant lower likelihood of reporting fear of side effects as a reason for refusing vaccination (column 1). The fear of pain from vaccination tended to be lower among older women who were unwilling to get vaccinated, but the difference was significant only for women aged 30-39 (column 2). Older women who refused vaccination were in turn significantly more likely to doubt the effectiveness of the COVID-19 vaccines than younger women who were also unwilling to get vaccinated (column 3). Women in age group 30-39 were further weakly significantly more likely to state a lack of belief in vaccines as a reason for not getting vaccinated, while tertiary or higher education was associated with a significantly lower likelihood of reporting this reason (column 4). Regarding the risk assessment of COVID-19, women whose households were situated in the middle or the highest wealth tertile were significantly less likely to state that they did not consider themselves to be at risk of COVID-19 (column 5). None of the examined socio-demographic characteristics were significantly associated with the likelihood of stating lack of a vaccine of choice or lack of information on vaccines as reasons for being unwilling to get vaccinated (columns 6 and 7). The likelihood of stating that negative information in the media had led to the refusal of vaccination against COVID-19 was significantly lower among women with tertiary or higher education, among women living in urban areas, and among women in age group 40-49, with the latter difference only being weakly significant, while higher relative household wealth was significantly associated with a higher likelihood of basing vaccine refusal on negative media information (column 8). Citing religious opposition as a reason for refusing vaccination was significantly and negatively associated with belonging to the middle tertile of household wealth; affiliation to Muslim or Pentecostal religion was not significantly associated with stated religious opposition to vaccination (column 9).

**Table 5:** A multivariate linear regression showing factors associated with reasons for being hesitant about COVID-19 vaccination among women in Uganda

	(1)	(2)	(3)	(4)	(5)	(6)	(7)	(8)	(9)
	Fear of side effects	Fear of pain	Doubt effectiveness	No belief	Not at risk	No vaccine of choice	Lack of info	Negative info in media	Religion
**Age**									
Age 15-19	omitted	omitted	omitted	omitted	omitted	omitted	omitted	omitted	omitted
	(.)	(.)	(.)	(.)	(.)	(.)	(.)	(.)	(.)
Age 20-29	0.142	-0.058	0.001	0.007	0.009	0.004	0.010	0.026	-0.014
	(0.017)	(0.135)	(0.981)	(0.816)	(0.740)	(0.622)	(0.836)	(0.660)	(0.253)
Age 30-39	0.087	-0.074	0.170	0.078	0.036	0.013	-0.022	-0.005	0.006
	(0.187)	(0.046)	(0.007)	(0.051)	(0.285)	(0.252)	(0.686)	(0.940)	(0.801)
Age 40-49	0.238	-0.040	0.142	0.062	0.022	-0.001	-0.056	-0.122	-0.009
	(0.000)	(0.419)	(0.075)	(0.267)	(0.540)	(0.921)	(0.317)	(0.057)	(0.639)
Primary education or less	omitted	omitted	omitted	omitted	omitted	omitted	omitted	omitted	omitted
	(.)	(.)	(.)	(.)	(.)	(.)	(.)	(.)	(.)
Any secondary education	-0.073	-0.038	0.041	-0.049	0.034	-0.008	-0.011	-0.068	-0.005
	(0.217)	(0.260)	(0.485)	(0.130)	(0.273)	(0.153)	(0.827)	(0.229)	(0.760)
Tertiary or higher education	-0.109	-0.035	0.028	-0.068	-0.011	-0.004	-0.054	-0.190	-0.020
	(0.274)	(0.511)	(0.771)	(0.049)	(0.715)	(0.624)	(0.462)	(0.013)	(0.352)
Lowest wealth tertile	omitted	omitted	omitted	omitted	omitted	omitted	omitted	omitted	omitted
	(.)	(.)	(.)	(.)	(.)	(.)	(.)	(.)	(.)
Middle wealth tertile	-0.107	0.009	0.006	0.043	-0.070	-0.003	-0.044	0.167	-0.029
	(0.061)	(0.803)	(0.915)	(0.267)	(0.038)	(0.758)	(0.374)	(0.002)	(0.044)
Highest wealth tertile	0.020	-0.026	0.049	0.014	-0.119	-0.003	-0.073	0.224	-0.015
	(0.769)	(0.515)	(0.506)	(0.698)	(0.000)	(0.741)	(0.198)	(0.001)	(0.553)
Rural	omitted	omitted	omitted	omitted	omitted	omitted	omitted	omitted	omitted
	(.)	(.)	(.)	(.)	(.)	(.)	(.)	(.)	(.)
Urban	0.043	0.011	-0.078	-0.026	0.026	-0.003	0.023	-0.130	0.022
	(0.472)	(0.763)	(0.219)	(0.364)	(0.333)	(0.553)	(0.612)	(0.035)	(0.157)
Not Muslim									omitted
									(.)
Muslim									0.047
									(0.226)
Not Pentecostal									omitted
									(.)
Pentecostal									0.036
									(0.104)
N	584	584	584	584	584	584	584	584	522

Note: *P*-values reported in parentheses.

Source: Author’s own computation

## Discussion

### Consistency and interpretation of the results

Adding together the female respondents who have already been vaccinated against COVID-19 and those who were unvaccinated but willing to get vaccinated, the analysis of the PMA Uganda Phase 2 survey indicates a COVID-19 vaccine acceptance rate of more than 85% in late 2021. This high rate is consistent with a COVID-19 vaccine acceptance rate of around 90% reported in high-frequency phone surveys conducted in Uganda between 2020 and 2022 [18].

The results of this study further indicate a positive and significant age gradient in the uptake of COVID-19 vaccination among Ugandan women. This may reflect the consequences of a prioritization of COVID-19 vaccination for older individuals, coupled with awareness of the risk of severe COVID-19 increasing with age [19, 20]. However, the quantitative differences between the age groups were rather small. Recall that the sample only included individuals below age 50; a potentially steeper age gradient among older women could therefore not be investigated. The positive age gradient in vaccine uptake was furthermore complemented by a negative age gradient in vaccine refusal, albeit with the latter being less steep and not significant for all age groups.

The results also highlight a positive, significant and quantitatively large association between tertiary or higher education and the uptake of COVID-19 vaccination. There could be several reasons behind this finding: Women with higher education might work in professions that have been prioritized to receive the COVID-19 vaccines. Higher education might also be associated with higher trust in vaccines and hence a higher willingness of accepting a COVID-19 vaccine if it has been offered. Notably, the positive association between higher education and COVID-19 vaccination was not mirrored in a negative association between higher education and COVID-19 vaccine refusal. A straight-forward explanation for this pattern is that those highly educated women who have already been vaccinated represented a selected group from all the women who were willing to be vaccinated if given the opportunity. Hence, higher education was positively associated with the likelihood to have been vaccinated for women who were willing to get vaccinated; it was not associated with whether women were willing to be vaccinated or not. The role of education for attitudes towards vaccines therefore remains variable across studies, with recent results from Kenya reporting a negative association between education and COVID-19 vaccine refusal [21] and results from across five sub-Saharan African countries reporting a positive association [18].

Interestingly, while there was no household wealth gradient in COVID-19 vaccine uptake, both female economic activity and autonomy, as measured in terms of female labor supply and savings, were positively associated with female uptake of COVID-19 vaccination. This may imply that having their own economic resources at their disposal provided Ugandan women with greater agency to get vaccinated than women who did not have such means. In turn, recent and severe income losses to the women’s households appeared then to negatively affect women’s agency to get vaccinated. Women who were more economically active may have also placed greater importance on getting vaccinated in order to lower their risk of economic losses from COVID-19 infection than women who were less economically active.

Taken together, the positive associations of female education and economic agency with vaccine uptake can be put into the context of female economic empowerment being positively associated with female agency over health decisions and health outcomes [22–24]. From this perspective, the absence of an association between household wealth and female vaccine uptake is in turn not surprising, as greater household wealth does not necessarily translate into greater female empowerment.

The positive association between vaccine uptake and the usage of modern contraceptives may further imply that users of modern contraceptives were more trusting of modern pharmaceuticals including the COVID-19 vaccines than non-users. Further, women who procured modern contraceptives may have had better access to health infrastructures which have potentially also provided COVID-19 vaccinations.

Refusal to be vaccinated against COVID-19 was significantly more prevalent among women who were pregnant. This may point to concerns among pregnant Ugandan women related to the safety of COVID-19 vaccines, complementing international evidence of elevated refusal of vaccination among pregnant women [25] and potentially indicating insufficient public health information distribution on the safety profile of the COVID-19 vaccines during pregnancy.

Overall, refusal of vaccination against COVID-19 among Ugandan women exhibited only few strong and significant associations with the socio-demographic characteristics considered in this study. Similarly, from the investigation into female respondents who have declared their unwillingness to be vaccinated against COVID-19, it appeared that socio-demographic characteristics had only little explanatory power for why vaccine-refusing individuals stated a particular reason for their refusal. Among the potential drivers of vaccine refusal not included in the PMA Uganda Phase 2 survey, the literature suggests that trust in its various forms is a strong predictor of vaccine refusal, also in African countries [21, 26].

Among the reasons for refusing vaccination, the fear of side effects stood out, as it was selected by more than two-thirds of the women who were unwilling to get vaccinated. Again, this finding is consistent with [18] reporting high rates of concerns about vaccine side effects and vaccine safety among the vaccine-hesitant in Uganda and other sub-Saharan African countries. While it should be kept in mind that the high prevalence of fear of side effects applied only to the relatively small share of women who refused vaccination, the significant increase in fear of side effects among the oldest age group considered here is concerning due to the risk of severe COVID-19 likewise increasing with age.

Regarding potential spatial gradients in COVID-19 vaccine uptake and refusal, urban residence was positively associated with both vaccine uptake and refusal in comparison to rural residence. While this finding may point to advantages for female urban residents in obtaining vaccination, it also points to heterogeneity within locations regarding attitudes towards vaccination.

### Limitations

The focus of the PMA survey on the female population precluded investigations into COVID-19 vaccine uptake and refusal among Ugandan men. The evidence presented in this study therefore needs to be complemented with comparable data on male or mixed populations. The focus on women aged 15-49 further excluded the older female population, which is at higher risk of severe COVID-19 than the survey population. However, UNFPA calculates that only 2% of Uganda’s current population are at age 65+ [27]; the uptake of the COVID-19 vaccines among the sample population is therefore closely aligned with the uptake among the overall adult population of Uganda.

While the PMA survey provides a broad range of socio-demographic variables, there is only limited information on the health status of the surveyed women. Hence, it was not possible to assess to what extend women with comorbidities associated with elevated risk of severe COVID-19 have been vaccinated against COVID-19.

Importantly, the associations of the various factors with COVID-19 vaccine uptake and refusal that are presented in this study may not be interpreted as causal relationships, as unobserved factors may exist that are correlated with both the observed socio-demographic variables and the vaccination outcomes and attitudes.

## Conclusion

Following the approval of the first COVID-19 vaccines, global efforts were directed at offering vaccination against COVID-19 to everyone who was willing to be vaccinated within a reasonable time frame. According to a representative survey collected among Ugandan women aged 15-49 between September and November 2021, this goal had not been reached yet by that time, as three quarters of the respondents in the sub-Saharan African country stated a willingness to be vaccinated against COVID-19 that could not have been translated into actual vaccinations yet. Given that deliveries of COVID-19 vaccines to African countries were still constrained by booster campaigns in vaccine-manufacturing countries by that time, calls for increasing vaccine manufacturing capacities in African countries for future health crises seem warranted [28–30].

By the time of the survey collection, the low vaccine uptake was further not distributed uniformly across the female survey population, as age and in particular higher education were positively and significantly associated with the probability of being vaccinated. These findings may reflect the consequences of earlier prioritizations of the access to the vaccines due to restricted supplies. Given that Uganda’s COVID-19 vaccination rate has been rising considerably since the survey collection, it is possible that these gradients have weakened in the meantime and a considerable share of the gap between the willingness to get vaccinated and the availability of vaccines has been closed. However, the global rise and subsequent dominance of the various Omicron variants shortly after the completion of the PMA survey collection may in turn have negatively impacted the inclination of Ugandans to be vaccinated, as the population has been largely exposed to the virus irrespective of the immunization coverage.

While less than one-sixth of the female respondents stated an unwillingness to be vaccinated against COVID-19 if a vaccine were offered to them, this unwillingness only correlated sparsely with socio-demographic characteristics of the respondents; even less so with regard to the particular reasons for being unwilling to be vaccinated. Consequently, the analysis suggests only few angles from which public health actors and institutions could address vaccine hesitancy and refusal in Uganda. One group that could benefit from targeted information provision on the safety profiles of the COVID-19 vaccines are pregnant women, who exhibited a significantly higher rate of vaccine refusal. Further, the pronounced fear of side effects that was showing up among the vaccine-refusing respondents in general suggests that public health authorities may need to consider aligning their provision of information on the COVID-19 vaccines accordingly. In this context, experimental interventions testing the effectiveness of public health-related messages for reducing vaccine hesitancy could be expanded to African countries in order to guide future public health messaging [31].

## Data Availability

The dataset supporting the conclusions of this article is available in the PMA data repository upon registration, URL: http://doi.org/10.34976/wf58-4e96.

## Abbreviations

BiB: Bundesinstitut für Bevölkerungsforschung
COVID-19: Coronavirus disease-2019
DHS: Demographic and Health Survey
OxCGRT: Oxford COVID-19 Government Response Tracker
PMA: Performance Monitoring for Action
